# Influence of Molybdenum in Drinking Water or Feed on Copper Metabolism in Cattle—A Review

**DOI:** 10.3390/ani11072083

**Published:** 2021-07-13

**Authors:** Meghan P. Thorndyke, Octavio Guimaraes, Maddie J. Kistner, John J. Wagner, Terry E. Engle

**Affiliations:** 1 Department of Animal Sciences, Colorado State University, Fort Collins, CO 80523-1171, USA; meghan.thorndyke@colostate.edu (M.P.T.); octavio.de_almeida_guimaraes_bisneto@colostate.edu (O.G.); john.wagner@colostate.edu (J.J.W.); 2 Compass Nutrition, Stephenville, TX 76401, USA; mkistner72@aol.com

**Keywords:** apparent absorption, apparent retention, rumen bypass, ruminant

## Abstract

**Simple Summary:**

The majority of research examining the antagonist impact of molybdenum (Mo) on copper (Cu) absorption and metabolism in ruminants has been conducted by supplementing dietary Mo, alone or in combination with elevated dietary sulfur (S) concentrations, and monitoring the Cu status of the animals. However, little research has been conducted investigating the impact of Mo in water on Cu metabolism in ruminants. Based on the limited number of experiments available for this review, it appears that Mo in drinking water may have a lower antagonistic impact on Cu status in cattle when compared to Mo consumed in the diet. Therefore, this review will focus on the impact of Mo in drinking water on cattle performance and Mo and Cu metabolism.

**Abstract:**

The majority of Mo research has focused on the antagonist effect of Mo, alone or in combination with elevated dietary S, on Cu absorption and metabolism in ruminants. Diets containing both >5.0 mg of Mo/kg DM and >0.33% S have been reported to reduce the Cu status in cattle and sheep. Therefore, due to the potential for inducing Cu deficiency, Mo and S concentrations in the diet should be monitored and kept within appropriate values. Elevated sulfate concentrations in drinking water can also be detrimental to livestock production, especially in ruminants. High concentrations of sulfate in water have been extensively studied in cattle because high-sulfate water induces polioencephalomalacia in ruminants. However, little research has been conducted investigating the impact of Mo in water on Cu metabolism in ruminants. Based on the limited number of published experiments, it appears that Mo in drinking water may have a lower antagonistic impact on the Cu status in cattle when compared to Mo consumed in the diet. This response may be due to a certain percentage of water bypassing the rumen when consumed by ruminants. Therefore, the objective of this review was to examine the impact of Mo in drinking water on cattle performance and Mo and Cu metabolism.

## 1. Introduction

The impact of water quality on beef cattle production has been extensively studied. Common water quality contaminants that negatively impact livestock production have been reported in the literature and have been summarized [1,2]. These include microbial contamination, elevated soluble salts, nitrates, sulfates, and soluble cations such as calcium (Ca) and magnesium (Mg). Other elements such as copper (Cu), manganese (Mn), and zinc (Zn) can also be detrimental to water quality. However, these elements are not typically found in concentrations great enough to impact water consumption or animal performance.

Certain areas within the Rocky Mountain region contain natural deposits of rock and/or sediment that present high concentrations of molybdenum (Mo). Due to natural geological events or human activity, Mo deposits can become soluble and enter ground or surface water systems [3]. In 2012, the Environmental Protection Agency set a Colorado Mo agricultural water standard of 160 μg/L. Although the impact of elevated water concentrations of Mo on livestock production has not been extensively studied, the influence of dietary Mo concentrations on livestock production in conjunction with dietary sulfur (S) has been extensively investigated. In ruminants, dietary concentration of Mo (ranging from 4 to 50 mg Mo/kg DM) have been reported to reduce the Cu status in cattle and sheep [4,5,6]. However, elevated dietary Mo concentrations in combination with elevated dietary S (>0.33%) concentrations have been reported to substantially reduce the Cu status in cattle and sheep [7]. This is most likely through the formation of thiomolybdates in the gastrointestinal tract of the animals [8,9,10,11,12]. Because Cu is an essential dietary element involved in numerous biochemical reactions in ruminants [13,14], understanding the impact of elevated Mo and/or S concentrations on Cu absorption and tissue deposition is important.

Due to the negative impact of high dietary concentrations of Mo (>5.0 mg Mo/kg DM), alone and, especially, in combination with elevated dietary S concentrations, on the Cu status in ruminants, the Mo and S concentrations of water should be considered when formulating diets for ruminants to prevent Cu deficiency and optimize animal performance. The impact of high S concentrations in water on animal performance is beyond the scope of this review. For in-depth reviews of the impact of S in water and diets on livestock production, see Wright [1] and Drewnoski et al., [15], respectively. This review will focus on the impact of Mo in drinking water on cattle performance and Mo and Cu metabolism.

## 2. Molybdenum

### 2.1. Soil

Molybdenum is typically found in soils as sesquioxide, water-soluble Mo or organically bound Mo [16]. The availability of Mo in soil to the plant depends on soil pH and on the concentration of other elements such as phosphate [16,17]. In acidic soils, Mo is mainly in sesquioxide forms (e.g., iron oxides) and thus unavailable for plant uptake [16] or entry into water systems as soluble Mo. Soils with an alkaline pH (e.g., soils that are poorly drained and aerated) have typically greater concentrations of water-soluble Mo. which is available for uptake by plants and possible movement into water systems [18]. Therefore, the total Mo soil concentration is not a good indicator of Mo concentrations in the vegetation.

### 2.2. Plants

In general, Mo concentrations in the vegetation used for livestock production typically range from 0.6 to 3.5 mg Mo/kg DM but have been reported to be in excess of 50 mg Mo/kg DM in areas with alkaline soil and elevated soil Mo concentrations [2,16,17,19]. Molybdenum absorption in plants is mostly from soluble forms of Mo in the soil, predominantly molybdate (MoO_4_^−2^; [20]). Molybdenum plays a functional role in nitrogenase in plants. Nitrogenase functions to convert nitrogen to ammonia, which is then used by plants [21]. Molybdenum also works in nitrate reduction as an electron carrier in the enzyme nitrate reductase. Nitrate reductase reduces nitrate to nitrite in plants and in some microbial populations associated with plant roots and within the gastrointestinal tract of animals [22]. Reddy [23] reported that the application of Mo to the soil can increase the crude protein content of plants.

Due to the role of Mo in nitrogen fixation in plants, Mo deficiency can cause a reduction in plant nitrogen metabolism and overall nitrogen fixation [20]. Legume plants such as ladino clover and birdsfoot trefoil have a higher Mo requirement than non-legume plants. Legumes grown in alkaline soils (pH 7.0–7.5) are the most efficient in absorbing Mo [24], most likely because Mo becomes soluble at alkaline pH. Although little data are available as to what is deemed normal for Mo concentrations in soil, normal soil Mo concentrations are suggested to range from 0.5 to 5 mg Mo/kg DM [20]. Legumes grown in areas with naturally high concentrations of Mo in the soil have been found to contain between 20 and 40 mg of Mo/kg DM [25].

In a three-year plant study, Jensen and Lesperance [26] compared Mo accumulation by several forage plant species with variables indicative of plant growth. They reported that Mo accumulation by plants typically used as feed for grazing livestock was affected by soil pH, plant species, location within the plant, depth to the water table, and agronomic practices. Other researchers have confirmed the findings of Jensen and Lesperance [19,26,27]. Overall, Mo accumulation in plant tissues, in the aforementioned experiments, varied significantly. Legumes generally accumulated more Mo than grasses, likely due to the role of Mo in nitrogen fixation in legumes [19,26,28], although there was a large variation in Mo concentrations between individual plant species [19,25]. The Mo content of legumes and grasses appeared to increase with the age of the plant [17,25]. Growing plants are able to absorb soluble Mo from an alkaline soil, and Mo concentration appears to be the greatest in the blades and leaves of plants (in concentrations of 1.5 to 5.0 mg Mo/kg DM, which may impact Cu metabolism in grazing ruminants) [17,25]. Forage grown where the water table was near the soil surface contained more Mo than forage grown on soil with a greater water depth [26]. Concentrations of Mo in plants ranged from values indicative of plant Mo deficiency (<0.10 mg Mo/kg DM) to values as high as 300 to 400 mg Mo/kg DM [19].

### 2.3. Water

Water is frequently suggested as a source of excess Mo, although no cases of Mo toxicity due to consumption of Mo in water have been confirmed under practical production conditions. Since cattle consume much larger quantities of water than dry matter, the expectation of Mo toxicity (diarrhea, anorexia, weight loss, stiffness, changes in hair color) under lower water Mo concentrations would be logical [29]. For approximate water intakes for beef cattle, see Table 1, adapted from the NASEM [2]. Estimates of water consumption are related to cattle production classification, body weight, and ambient temperature. Numerous factors, such as dry matter intake, animal species, breed, stage of production, water quality, and the environment can influence water intake. Table 2 shows the calculated influence of dietary and water Mo concentrations on total Mo consumed (mg) per day for different dietary and water Mo concentrations. Based on these calculations, assuming that a mature beef animal would consume approximately 50 L of water and 10 kg of dry matter per day, if water and feed Mo concentration were equal, water would contribute 5 times the amount of total Mo intake with respect to feed.

### 2.4. Functions

Molybdenum functions as a component of several oxidase enzymes in animals (xanthine oxidase, sulfite oxidase, and aldehyde oxidase) [32,33]. Numerous reviews have been published on the impact of dietary Mo on ruminants’ Cu status and overall production [2,13,14,29,33,34,35,36,37,38]. However, as mentioned previously, the dietary requirements for Mo are not well defined for beef cattle because, under practical feeding conditions, Mo deficiency has not been reported [2,33]. There is limited evidence that the addition of Mo to sheep and beef cattle diets may improve diet digestibility depending on diet type (roughage compared to concentrate). Adding 10 mg of Mo/kg diet DM to a high-fiber diet (basal diet contained 1.7 mg of Mo/kg DM) improved in situ DM digestibility in steers compared to control animals but had no impact when animals were fed a ground barley-based diet supplemented with Mo [39]. Earlier research by Ellis et al. [40] reported an improvement in growth rate and cellulose digestion in lambs supplemented with 2 mg of Mo/kg DM (basal diet contained 0.36 mg Mo/kg DM). However, Ellis and Pfander [41] were unable to reproduce this improvement in growth and cellulose digestibility in subsequent experiments.

Arguably, the most extensively studied aspect of Mo in ruminant diets is in relationship to dietary S and Cu concentrations. Ferguson et al. [42] originally reported that excess Mo in the form of ‘teart’ herbage (herbage with elevated Mo concentrations) caused: (1) a decrease in milk production, (2) scours, and (3) a reduction of body condition in grazing cattle. Ferguson et al. [42] were unsure if Mo was directly responsible for the symptoms reported or if it was metabolized to a toxic product in the rumen. Dick and Bull [4] were the first to report that long-term Mo supplementation reduced the Cu status in cattle. Later, Dick [43] reported similar impacts of elevated dietary Mo on reducing the Cu status in sheep. Upon further investigation, Dick [8] determined that the dietary S content substantially increases the antagonistic impact of Mo on Mo and Cu metabolism in sheep. Since this discovery, several researchers have examined the impact of dietary Mo and S concentrations on Cu metabolism in cattle and sheep [6,10,11,44]. Collectively, these data indicate that elevated dietary concentrations of Mo (≥5.0 mg Mo/kg DM) in the presence of adequate dietary S concentrations (≈0.2% S) or elevated dietary S (≥0.33 % S) concentrations in the presence of moderate Mo (<2.0 mg Mo/kg DM) concentrations can modestly reduce Cu absorption in ruminants by 0.5 to 5%. This antagonism is most likely through the formation of insoluble Cu–Mo or Cu–S complexes. The location of the formation of Cu–Mo and Cu–S complexes in the gastrointestinal track is not completely understood [11]. Additionally, elevated dietary S (≥0.33% S) in the presence of elevated dietary Mo (≥5.0 mg Mo/kg DM) concentrations can drastically reduce Cu absorption in cattle and sheep by approximately 60%. The reduction in Cu status with elevated dietary Mo and S is most likely due to the formation of insoluble Cu–Mo–S complexes in the rumen. For extensive reviews of the formation and impact of thiomolybdates on Cu metabolism in ruminants, see [12,14,38].

### 2.5. Dietary Molybdenum and Copper

Molybdenum toxicity can be of concern due to possible depletion of Cu stores through the formation of thiomolybdates. For a thorough review of the formation of thiomolybdates in ruminants, see Suttle [12]. A physiological Cu deficiency in animals from excess Mo consumption can lead to a multitude of issues affecting growth and overall animal health. Molybdenum intake in feed has been closely studied. In 1975, Kubota [45] examined the geographic distribution of Mo in western states of the USA in relation to historical reports of molybdenosis and Cu deficiency in grazing beef cattle. Kubota [45] concluded that cattle grazing forages with 10 to 20 mg of Mo/kg DM were likely to exhibit Mo toxicity symptoms. In a review by Underwood and Suttle [14,42], cattle grazing teart pastures that contained between 20 and 100 mg of Mo/kg plant DM experienced mild to extreme forms of scouring [42].

The Cu/Mo ratio is important, as well as the total concentration of Mo in the diet. Ward [29] reviewed previously published data investigating the influence of Mo dietary intake on cattle performance and Cu status. Ward [29] concluded that cattle fed more than approximately 100 mg of Mo/kg DM or fed forage with a Cu/Mo ratio of 2:1 or less can experience Cu deficiencies. Gardner et al. [46] investigated the impact of Mo on gestating cows nursing calves and grazing standing forages containing between 21 and 44 mg of Mo/kg DM in reclaimed mining areas. Cow–calf pairs were allowed to graze for 12 weeks each year for three consecutive years on the reclaimed mining area pastures. No signs of molybdenosis, Cu deficiency, or any adverse health effects, regardless of whether cattle received a Cu bolus or not, were reported. The authors concluded that the risk assessment value of 10 mg of Mo/kg DM reported by O’Conor et al. [47] to establish Mo standards for land application of biosolids is conservative for grazing cattle. However, the reason for the lack of observed molybdenosis in these cattle may be the short duration of Mo exposure in the Gardner et al. [46] experiment.

In 2006, Raisbeck et al. [48] conducted a long-term grazing experiment where pregnant cows (*n* = 306) were grazed on one of three pastures containing different Mo concentrations in the standing forage (2.0, 13, and 230 mg of Mo/kg DM for pastures 1, 2, and 3, respectively). Pasture 1 contained 59 cows, pasture 2 contained 241 cows, and pasture 3 contained 6 cows. For pastures 2 and 3, all cattle were given a protein supplement formulated to provide 17 mg of Cu/kg DM total diet. Furthermore, half for the cows in pastures 2 and 3 received a 25 g CuO bolus (21.8 g elemental Cu equivalent) every 60 d throughout the 12-month experiment. Cattle in pasture 1 received protein supplementation but minimal Cu supplementation (2 mg Cu/kg DM total diet), and no CuO boluses were administered to these cows. Although not described fully in the publication, Raisbeck et al. [48] indicated that standing forage Cu concentrations varied across season and that cattle were fed supplemental alfalfa hay during the winter months. Therefore, the approximate Cu/Mo ratios for cattle in pasture 1 and cattle not receiving CuO boluses in pastures 2 and 3 were 3.7:1, 1.8:1, and 0.10:1, respectively. Raisbeck et al. [48] reported no adverse effects of Mo on cattle housed in pastures 1 and 2 and noted that two cattle in pasture 3 (230 mg Mo/kg DM in standing forages) exhibited diarrhea and lameness (signs indicative of molybdenosis) in the last two weeks of the experiment. However, the bull was diagnosed with a spinal injury and treated with flunixin meglumine, and the cow was diagnosed with a bacterial infection and treated with an antibiotic. Both animals recovered within a week of being treated and were physiologically normal. Raisbeck et al. [48] also reported that the Mo and Cu statuses were not impacted in cattle grazing pastures containing 13 mg of Mo/kg DM. However, cattle grazing pastures containing 230 mg of Mo/kg DM exhibited an increase in liver Mo concentrations as well as an increase in soluble serum Cu and liver Cu concentrations, regardless of CuO bolus administration. The authors concluded that cattle receiving the greatest Mo concentrations (230 mg Mo/kg DM) had an increased Mo status indicative of molybdenosis but never showed signs of molybdenosis and that no negative impacts on animal growth, calf weaning weighs, or reproductive performance in any of the animals in the experiment were observed. The Mo and Cu concentrations consumed by cattle in pasture 2 (13 mg Mo/kg DM) of the Raisbeck et al. [48] experiment were approximately 156 mg of Mo∙head^−1^∙d^−1^ and 240 mg of Cu∙head^−1^∙d^−1^ (using an estimated DMI of 12 kg∙head^−1^∙d^−1^, a Mo forage concentration of 13 mg Mo/kg DM, and an estimated total diet Cu concentration of 20 mg Cu/kg DM), the dietary Cu/Mo ratio was 1.8:1 and did not impact Cu status or animal performance.

### 2.6. Molybdenum and Water

As previously described, the majority of research investigating the impact of Mo on Cu metabolism in ruminants has been conducted by supplementing varying concentrations of dietary Mo and S and monitoring the Cu status of the animal. Very few studies have investigated the impact of Mo supplied in drinking water on Cu metabolism in ruminants.

In 1980, Kincaid [18] conducted an experiment utilizing 12 male, 5-week-old Holstein calves. Calves were allowed ad libitum access to drinking water containing targeted concentrations of 0.0, 1.0, 10.0, and 50.0 mg of Mo/L (analyzed Mo concentrations were <1.0, 1.0, 8.0, and 53.0 mg Mo/L, respectively) for 21 days. The basal diet for these calves contained 13 mg of Cu/kg DM (<1 mg of Mo/kg diet DM and 0.29% S). There was no difference in body weight gain across all treatments. At the greatest Mo water concentration (50.0 mg of Mo/L), Kincaid [18] reported an increase in plasma Cu concentrations and a numeric decrease in liver Cu concentrations. Calves receiving 0.0, 1.0, and 10.0 mg of Mo/L in drinking water had similar plasma and liver Cu concentrations and ceruloplasmin levels. Kincaid [18] indicated that the safe Cu-to-Mo ratio in this experiment was 0.5:1.0. Kincaid [18] also suggested that Mo in water could be less toxic than Mo in forage and that the minimum toxic concentration of Mo in water for calves in this experiment was between 10 and 50 mg of Mo/L.

In 2017, Kistner et al. [3] performed an experiment with 30 Angus, Hereford, and Angus × Herford steers exposed to varying doses of Mo in drinking water. Water treatments consisted of: (1) 0.0 mg/L, (2) 0.16 mg/L, (3) 0.32 mg/L, (4) 0.48 mg/L, and (5) 0.96 mg/L of supplemental Mo added as Na_2_MoO_4_ to drinking water. Steers were housed in individual pens and fed a growing diet for 28 days and then transitioned to a finishing diet. Mo exposure was maintained for a period of 112 to 151 days. No adverse effects were observed in any animals at any Mo dose. Total dietary Cu concentrations ranged from 9.7 to 11.1 mg of Cu/kg DM in order to meet the NASEM [2] guidelines of 10 mg of total Cu/kg diet DM. A Cu-to-Mo no-effects ratio (no impact on Cu status or growth performance) of 2.8:1 was measured in this experiment. Kistner et al. [3] hypothesized that Mo in drinking water may have a lower impact on the Cu status in cattle, possibly due to water bypassing the rumen when consumed.

Ruminal bypass of drinking water via the esophageal groove has been estimated to be between 18 and 80% of the water consumed by mature cattle [49,50,51,52,53]. The wide range of the proportion of drinking water that can bypass the rumen may be due to diet type. Garza et al. [53] estimated drinking water ruminal bypass (using two different markers, i.e., polyethylene glycol and chromium-EDTA) in cattle consuming a high-concentrate diet compared to cattle consuming a high-forage diet. The authors estimated that approximately 49% of the drinking water consumed bypassed the rumen when cattle were consuming a high-forage diet compared to 79% water bypass in cattle consuming a high-concentrate diet. Other factors such as the height of the water trough, drinking frequency and duration, and other dietary ingredients may also impact the ruminal bypass of drinking water.

Based on the findings of Kincaid [18] and Kistner, et al. [3], it appears that Mo in water may have a low impact on Cu metabolism in ruminants, possibly due a portion of the water consumed bypassing the rumen. If this theory is correct, a portion of Mo consumed through water would theoretically not be exposed to the reducing environment of the rumen and therefore be less available to form thiomolybdates. To test this hypothesis, Thorndyke et al. [54] utilized 12 Angus steers and investigated the influence of Mo in drinking water or feed on the apparent absorption and retention of Cu and Mo. Steers were fed a low-quality grass hay diet (basal diet: 6.4% CP; 0.12% S, 6.8 mg Cu/kg DM; 2.5 mg Mo/kg DM) for a period of 14 days. The steers were then housed in metabolism stalls, and total fecal and urine output were collected for 5 days. During the collection period, treatments consisted of: (1) control, no supplemental Mo, (2) 5.0 mg Mo/kg DM from sodium molybdate dihydrate (MoNa_2_O_4_·2H_2_O; Mo-diet), and (3) 1.5 mg Mo/L from MoNa_2_O_4_·2H_2_O delivered in drinking water (Mo-water). The control steers consumed 25 mg Mo/d while Mo-supplemented animals consumed 79.7 and 72.3 mg Mo/d (Mo-diet and Mo-water steers, respectively). Dry matter intake, DM digestibility, water intake, and Cu intake were similar across treatments. Mo intake was lower in controls compared to Mo-supplemented steers. Molybdenum-supplemented steers had lower apparent absorption and retention of Mo (when expressed as a % of Mo intake). However, the apparent absorption of Mo was similar between Mo-supplemented steers (Mo-diet and Mo-water). When expressed as mg Mo absorbed/d, Mo apparent absorption was greatest for steers receiving Mo-diet, followed by steers on Mo-water and control steers. The apparent retention of Mo (mg/d) was greater for Mo-diet steers when compared to Mo-water steers which had greater Mo apparent retention than control steers.

Urinary Cu excretion (mg/day) was lower in Mo-diet steers when compared to control and Mo-water steers. The apparent absorption of Cu (expressed as a % of Mo intake) was greater in controls compared to Mo-diet steers but similar to Mo-water steers. The percent apparent absorption of Cu tended to be lower in Mo-diet compared to Mo-water steers. When expressed as mg Cu/d, the apparent absorption of Cu was greater in control compared to Mo-water steers and in Mo-water than in Mo-diet steers. The apparent retention of Cu expressed as a percent of intake or mg/d was similar between control and Mo-water steers and different compared to Mo-diet steers. However, the apparent retention of Cu (expressed as either a % of Cu intake or mg Cu/day) was similar between Mo-water and Mo-diet steers. The results from Thorndyke et al. [54] indicate that Mo in water may have a lower impact on apparent absorption and retention of Cu than Mo in feed.

## 3. Conclusions

Based on the limited number of experiments available for this review, it appears that Mo in drinking water may have a lower antagonistic impact on Cu status in cattle when compared to Mo consumed in the diet. However, the experiment conducted by Kincaid [18] were performed on a small sample, had a short exposure period, and used young calves. Experiment with a longer in duration (≈5 months) were performed by Kistner et al. [3], who supplied Mo in drinking water to feedlot steers consuming a high-concentrate diet that contained 0.15% dietary S, whereas, Thorndyke et al. [54] examined an acute exposure of Mo in the water or diet over a 5-day period. In all experiments, dietary S concentrations were low to adequate. Future research should examine how diet type, duration of Mo exposure, and dietary and water S concentrations influence the impact of Mo supplied in the water on the Cu status of ruminants.

## Figures and Tables

**Table 1:** Influence of temperature, cattle type, and body weight on estimated daily water intake (L) for beef cattle ^a,b^.

Body Weight, kg	Temperature, °C ^b^					
	4.4	10.0	14.4	21.1	26.6	32.2
	**Growing Heifers, Steers, and Bulls**					
182	15.1	16.3	18.9	22.0	25.4	36.0
273	20.1	22.0	25.0	29.5	33.7	48.1
364	213.0	25.7	29.9	34.8	40.1	56.8
	**Finishing Cattle**					
273	22.7	24.6	28.0	32.9	37.9	54.1
364	27.6	29.9	34.4	40.5	46.6	65.9
454	32.9	35.6	40.9	47.7	54.9	78.0
	**Cows Pregnant/Lactating ^c,d^**					
409	24.4/43.1	27.3/47.7	31.4/54.9	36.7/64.0	NA ^e^/67.8	NA ^e^/61.3
	**Mature Bulls**					
636	30.3	32.6	37.5	44.3	50.7	71.9
727	32.9	35.6	40.9	47.7	54.9	78.0

^a^ Adapted from NASEM [2] and Winchester and Morris [30]. ^b^ Water intake of a given class of cattle in a specific management regime is a function of dry matter intake and ambient temperature. Water intake is quite constant up to 4.4 °C. ^c^ Dry matter intake has a major influence on water intake. Heavier cows are assumed to be higher in body condition and to require less dry matter and, thus, lower water intake. ^d^ Cows larger than 409 kg are included in this recommendation. ^e^ Data not available.

**Table 2:** Calculated influence of water molybdenum (Mo) concentrations and dietary molybdenum concentrations on total Mo intake, total mg of Mo consumed per day for beef cattle ^a^.

Diet Mo Concentration, mg Mo/kg DM	Water Molybdenum Concentration, mg/L ^b^				
	0.0	0.1	0.5	1.0	2.0
0.0	0.0	5.0	25.0	50.0	100.0
1.0	10.0	15.0	35.0	60.0	110.0
2.0	20.0	25.0	45.0	70.0	120.0
3.0	30.0	35.0	55.0	80.0	130.0
4.0	40.0	45.0	65.0	90.0	140.0
5.0	50.0	55.0	75.0	100.0	150.0
10.0	100.0	105.0	125.0	150.0	200.0
50.0	500.0	505.0	525.0	550.0	600.0

^a^ Adapted form Neuhold [31]. ^b^ Based on 50 L/animal daily water intake and 10.0 kg/animal dry matter intake.

## Data Availability

Data can be found in theses submitted to the Academic Faculty of Colorado State University in partial fulfillment of the requirements for the degree of Master of Science by M. P. Thorndyke: ISBN 9798556707816 and M. Kistner: ISBN 9781369869965.
